# Supplementary material for: Developmental seizures and mortality result from reducing GABAA receptor α2-subunit interaction with collybistin
Source: Nat Commun. 2018 Aug 7;9:3130. doi: 10.1038/s41467-018-05481-1 (PMC6081406; doi:10.1038/s41467-018-05481-1)
Supplement: Supplementary file 1 — Supplementary Information [file 41467_2018_5481_MOESM1_ESM.pdf]

Supplementary Information

Developmental seizures and mortality result from reducing $GABA_A$ Receptor α2 subunit interaction with collybistin

Hines et al.,

Supplementary Figures and Tables

Supplementary Table 1. Summary of collybistin SH3-GABA$_A$R binding parameters as determined by ITC. All values listed are mean ± standard error.

**Receptor**	**Stoichiometry** (N)	**K$_d$** (μM)	**ΔH** (kcal/mol)	**-TΔS** (kcal/mol)
GABA$_A$R-α1 WT	n.d.	500±400	-3±0.8	2.1
GABA$_A$R-α2 WT	0.62±0.03	1.3±0.8	-6.8±0.4	0.4

Supplementary Table 2. Truncated proteins used for ITC, showing their sequences and corresponding K$_d$ values.

	**Sequence**	**K$_d$** (μM)
α2-WT	NYFTKRGWAWDGKSVVNDKPKKEKGSVMIQNNAYAVAVANYAPNLSKDPVLSTISKSATTPEPNKKPENKPAEAKKTFNSVSKIDR	1
α2-351St	NYFTKRGWAWDGKSVVNDKPKKEKGSVMIQNNAYAVAVANYAPNL---	7
α2-min1	-----------------------------QNNAYAVAVANYAPN--	>500
α2-min2	-----------------------KGSVMIQNNAYAVAVANYAPNL---	>500
α2-min3	------------------KPKKEKGSVMIQNNAYAVAVANYAPNL---	>500
α2-335St	NYFTKRGWAWDGKSVVNDKPKKEKGSVMI---	>500

A

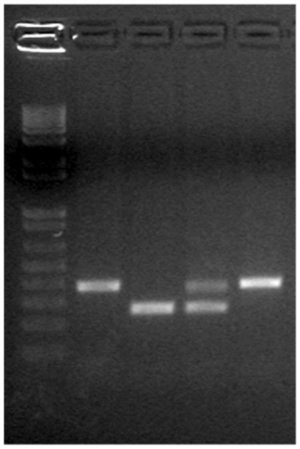

B

WT

```
NNNNNNNNNNNNNNNTNNGATTTACTGNCTTATTTTGCTTACTTCCTTAA
TTTTTCTCGCCTACTACTAATGATGCNTGTTTGAGTAATAATTTC
TTCTAGAATTTGAACTGTATTTTGGAAATTTTTAAGATGCAGCCT
AAACCCACATTGTGTCAACTTTTGTATTTTCCATTAGAAAAAAGA
GAAAGGCTCCGTCATGATACAGAACAACGCCTATGCGGTAGCTGT
TGCCAACTATGCCCCGAATCTTTCCAAAGATCCTGTCCTCTCTAC
CATTTCCAAAAGTGCAACGACGCCAGAACCGAACAAGAAGCCAGA
GAACAAGCCAGCTGAAGCCAAGAAAACCTTCAACAGTGTCAGCAA
AATCGACAGGATGTCTAGAATAGTGTTCCCAGTTCTGTTTGGTAC
TTTTAATTTNNTTTATTGGGCTACCTATTTAAACNNNNNNNNNNNNNNNN
```

Gabra2-1 Homo

```
NNNNNNNNNNNNNNNNNNGATTTACTGNNNTATTTTGCTTACTTCCTTAA
TTTTTCTCGCCTACTACTAATGATGCATGTTTGAGTAATAATTTC
TTCTAGAATTTGAACTGTATTTTGGAAATTTTTAAGATGCAGCCT
AAACCCACATTGTGTCAACTTTTGTATTTTCCATTAGAAAAAAGA
GAAAGATCCTCTCATTAAGAAAAACAACACATATGCTCCTACAGC
AACCAGCTATACCCCGAATCTTTCCAAAGATCCTGTCCTCTCTAC
CATTTCCAAAAGTGCAACGACGCCAGAACCGAACAAGAAGCCAGA
GAACAAGCCAGCTGAAGCCAAGAAAACCTTCAACAGTGTCAGCAA
AATCGACAGGATGTCTAGAATAGTGTTCCCAGTTCTGTTTGGTAC
TTTTAATTTAGTTTATTGGGCTACCTATTTAAACAGGGANNNNNNNNNNN
```

C

```
           WT  1 KKEKGSVMIQNNAYAVAVANYAPNLSKDPVLSTISKSATTPEPNKKPENK
                 ||||..::.:||.||....:|.||||||||||||||||||||||||||||
Gabra2-1 Homo  1 KKEKDPLIKKNNTYAPTATSYTPNLSKDPVLSTISKSATTPEPNKKPENK

           WT 51 PAEAKKTFNSVSKIDRMSRIVFPVLFGTFNXXYWATYLN
                 ||||||||||||||||||||||||||||||  |||||||
Gabra2-1 Homo 51 PAEAKKTFNSVSKIDRMSRIVFPVLFGTFNLVYWATYLN
```

D

```
α1 301 RNSLPKVAYATAMDWFIAVCYAFVFSALIEFATVNYFTKRGYAWDGKSVV  350
       |||||||||||||||||||||||||||||||||||||||||:||||||||
α2 301 RNSLPKVAYATAMDWFIAVCYAFVFSALIEFATVNYFTKRGWAWDGKSVV  350

α1 351 PEKPKKVKDPLIKKNNTYAPTATSYTPNLARGDPGLATIAKSATIEPKEV  400
       .:| ||.|..::.:||.||....:|.|||:: ||.|:||:||||......
α2 351 NDK-KKEKASVMIQNNAYAVAVANYAPNLSK-DPVLSTISKSATTPEPNK  398

α1 401 KPETKPPEPKKTFNSVSKIDRLSRIAFPLLFGIFNLVYWATYLNREPQL-  449
       |||.||.|.||||||||||||:|||.||:|||.||||||||||||||.|
α2 399 KPENKPAEAKKTFNSVSKIDRMSRIVFPVLFGTFNLVYWATYLNREPVLG  448
```

Supplementary Figure 1. Sequencing and genotyping confirmation of the *Gabra2*-1 mutation. A. PCR genotyping results demonstrating the banding pattern observed in *wildtype*, *heterozygous* and *homozygous* mice. B. Raw sequencing results from fragments amplified via PCR from *wildtype* or *Gabra2*-1 *homozygou*s mice. C. Predicted amino acid sequence and alignment from sequencing results comparing *wildtype* and *Gabra2*-1 *homozygous*. D. Alignment of $GABA_AR$-α1 and -α2 subunits centered on the large intracellular loop between transmembrane domains 3 and 4 showing the divergence in sequence.

Supplementary Table 3. Antibody information.

Antibody	Application	Dilution	Company / Source	Catalog
$GABA_AR$ α2 loop	Western blotting	1:1000	PhosphoSolutions	822-GA2CL
$GABA_AR$ α2 C-term	Western blotting	1:1000	PhosphoSolutions	No longer available
$GABA_AR$ α2	Immunocyto and immunohisto	1:500	Synaptic Systems	224 103
$GABA_AR$ α1	Immunohisto	1:500	UC Davis/NIH NeuroMab Facility	clone N95/35
VGAT	Immunohisto	1:1000	Millipore Sigma	AB2257
GAD-65	Immunohisto	1:1000	Synaptic Systems	198 104
CB	Western blotting and immunohisto	1:500	Synaptic Systems	261 003
Gephyrin	Western blotting and immunohisto	1:500	Synaptic Systems	147 021
Pan Na^+ Channel	Immunocyto and immunohisto	1:200	Millipore Sigma	S8809
Parvalbumin	Immunohisto	1:1000	Millipore Sigma	MABN1191
CB1R	Immunohisto	1:500	Synaptic Systems	258 104
Actin	Western blotting	1:50000	Millipore Sigma	A2228

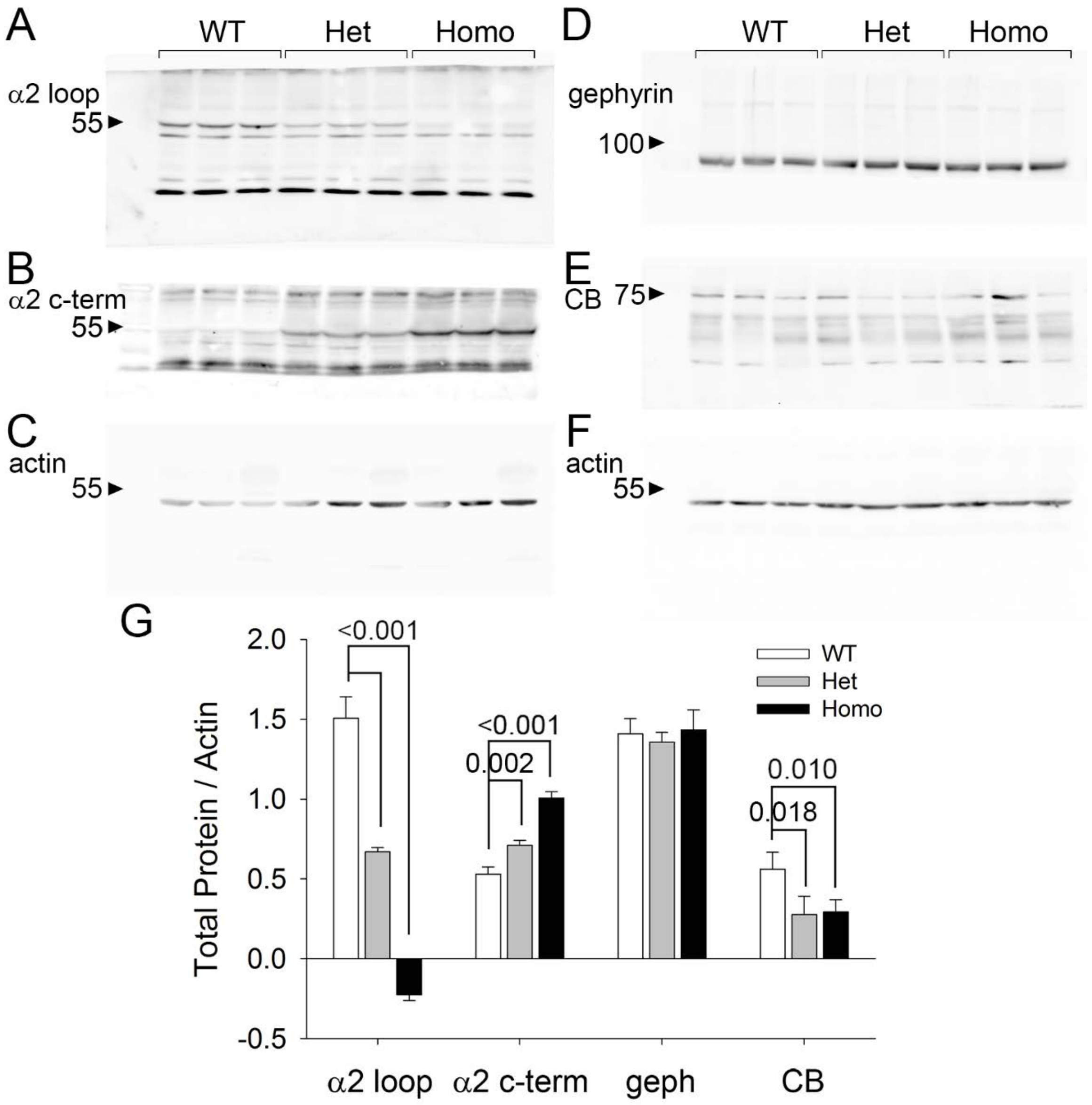

Supplementary Figure 2. Uncropped western blots and quantification of immunoblotting results in Ga*bra2*-1 mice. A-F. Representative images of $GABA_AR$-α2 loop (A), c-terminus (B), gephyrin (D), collybistin (E), and actin (C&F) immunoblotting. G. Quantification of expression of α2 loop (WT - 1.51 ± 0.061; Het - 0.67 ± 0.011; Homo - (-)0.23 ± 0.017), α2 c-term (WT - 0.53 ± 0.038; Het - 0.71 ± 0.026; Homo - 1.01 ± 0.033), gephyrin (WT - 1.41 ± 0.093; Het - 1.36 ± 0.061; Homo - 1.43 ± 0.126; ANOVA p=0.853), and collybistin (WT - 0.56 ± 0.068; Het - 0.28 ± 0.073; Homo - 0.29 ± 0.049) immunoblotting normalized to actin. All plots shown and all values listed are mean ± standard error.

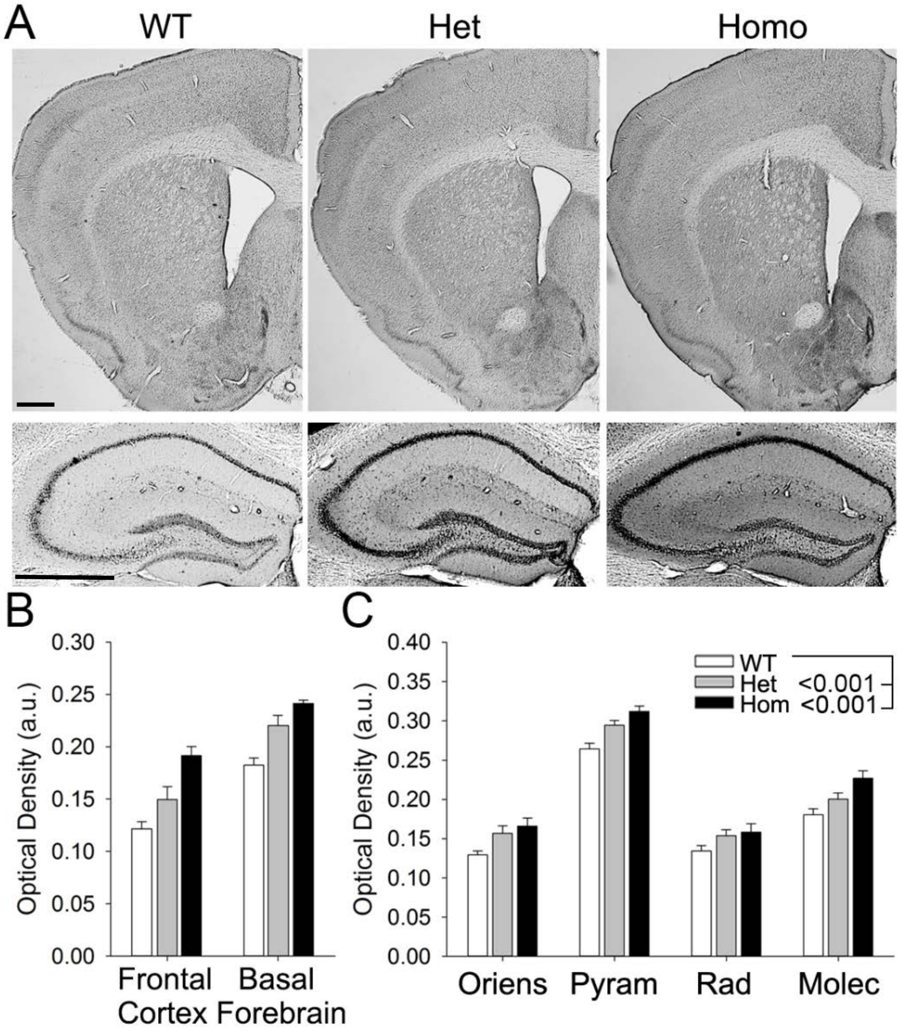

Supplementary Figure 3. Immunohistochemical localization of GABA$_A$R-α2 subunit in *wildtype* and *Gabra2*-1 mice. A. Representative images of GABA$_A$R-α2 subunit c-terminal staining comparing *wildtype* and *Gabra2*-1 tissue sections through the frontal cortex and basal forebrain (upper panels) and hippocampus (lower panels). B. Quantification of α2 staining intensity comparing *wildtype* and *Gabra2*-1 mice in frontal cortex and basal forebrain. C. Quantification of α2 staining intensity comparing *wildtype* (LS Mean 0.177), and *Gabra2*-1 *heterozygous* (LS Mean 0.201), and *homozygous* (LS Mean 0.216; SE of LS Mean – 0.00407) tissue in subregions of the hippocampus (Oriens LS Mean – 0.151; Pyramidal (Pyram) LS Mean 0.290; Radiatum LS Mean 0.149; Molecular (Molec) LS Mean 0.203; SE of LS Mean – 0.00407). Scale bars = 500 µm. All plots shown and all values listed are mean ± standard error.

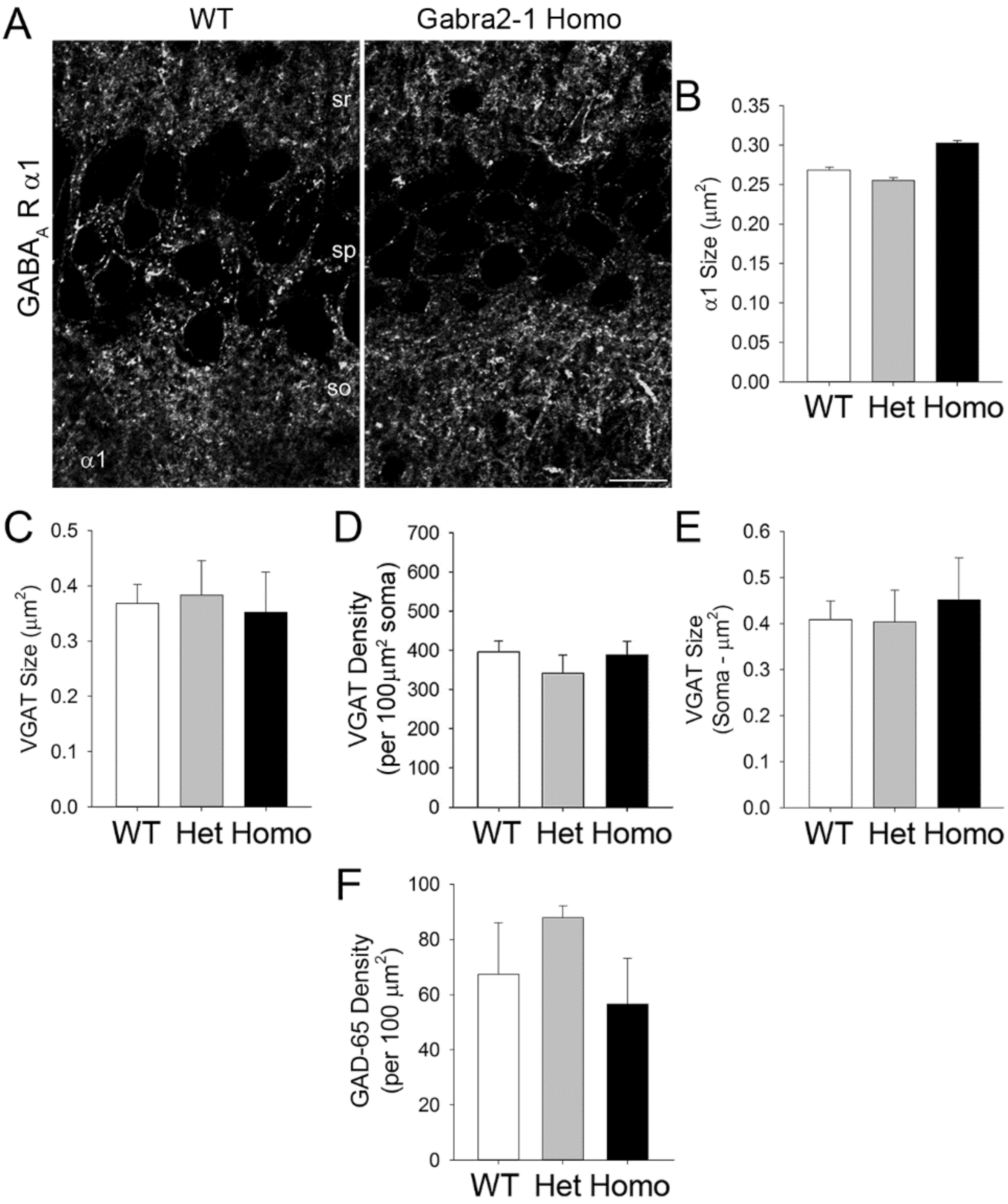

Supplementary Figure 4. GABA$_A$R-α1 subunit staining and additional IHC quantification for α1, VGAT, and GAD-65. A. Representative images of GABA$_A$R-α1 subunit staining comparing *wildtype* and *Gabra2*-1 *homozygous* CA1. Quantification of α1 cluster size (B), VGAT cluster size (C), VGAT cluster density on the soma (D), VGAT cluster size on the soma (E), and GAD-65 cluster density. Scale bar = 10 µm. All plots shown and all values listed are mean ± standard error.

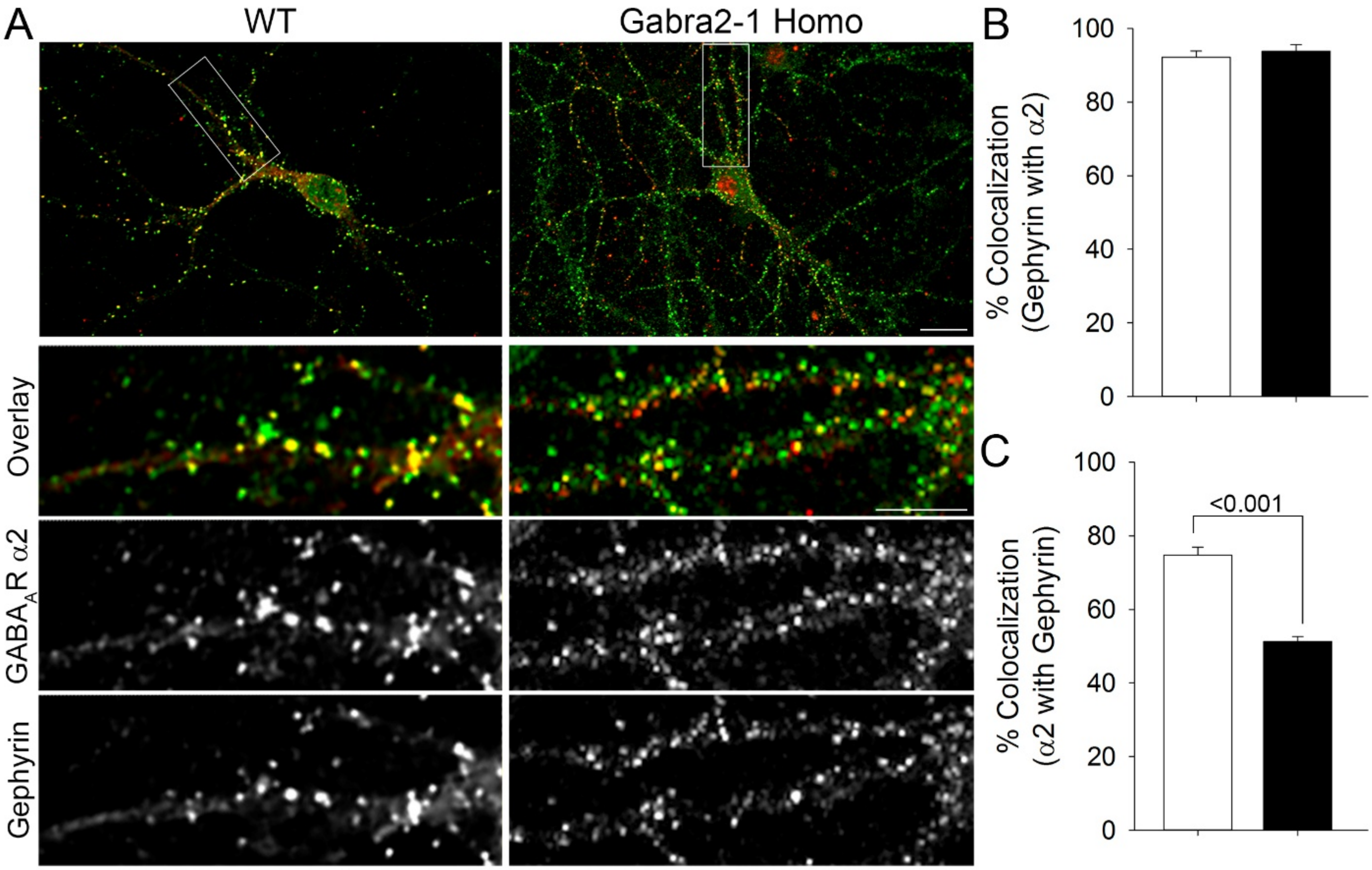

Supplementary Figure 5. Colocalization of gephyrin and GABA$_A$R-α2 subunit is maintained in cortical cells cultured from *Gabra2*-1 *homozygotes*. A. Representative images of GABA$_A$R-α2 subunit (green) and gephyrin (red) co-staining in cultured cortical neurons. B. Quantification of the percent colocalization of gephyrin with α2, comparing wildtype (92.18 ± 1.696) and *Gabra2*-1 *homozygous* (93.82 ± 1.743; p=0.322) cultures. C. Quantification of the percent colocalization of α2 with gephyrin comparing *wildtype* (74.76 ± 2.128) and *Gabra2*-1 *homozygous* (51.30 ± 1.331; p<0.001) cultures. Scale bar: top right overlay panel = 10 µm; lower right overlay panel = 5 µm. All plots shown and all values listed are mean ± standard error.

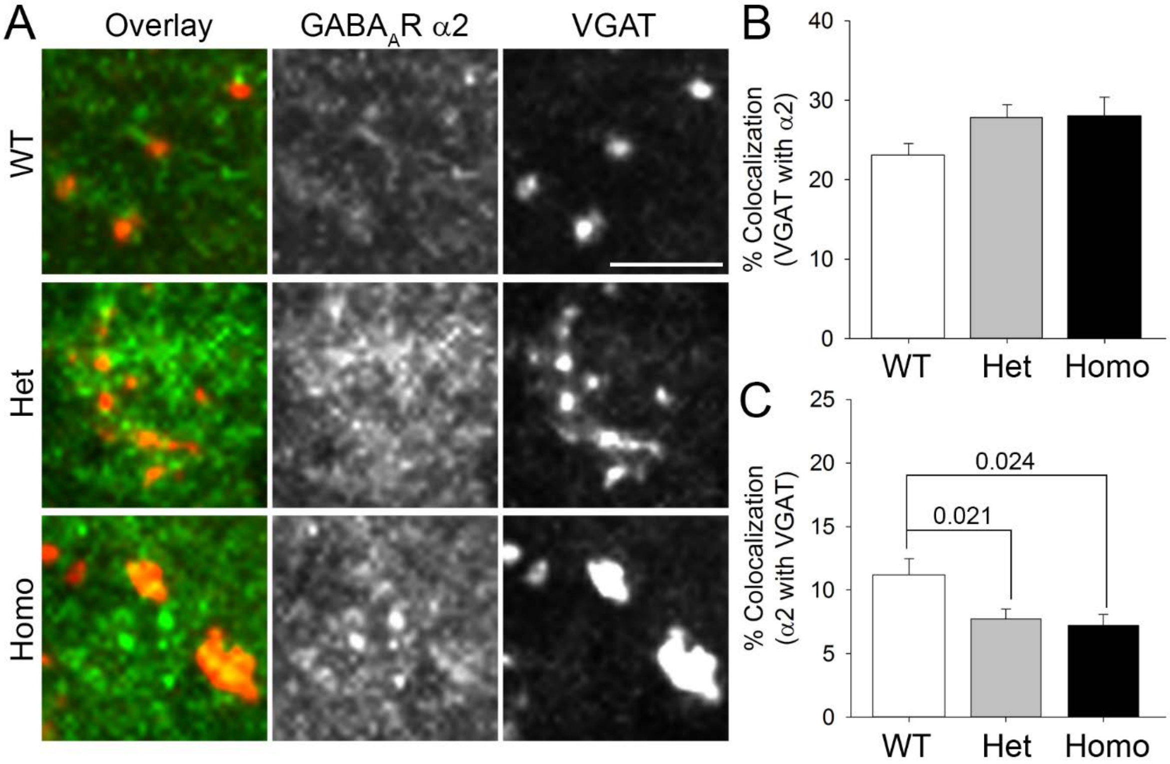

Supplementary Figure 6. Colocalization of VGAT and GABA$_A$R-α2 subunit is maintained in *Gabra2*-1 mice. A. Representative images of GABA$_A$R-α2 subunit (green) and VGAT (red) co-staining in frontal cortex. B. Quantification of the percent colocalization of VGAT with α2 comparing *wildtype* (23.12 ± 1.448), with *Gabra2*-1 *heterozygous* (27.84 ± 1.595) and *homozygous* (28.06 ± 2.313; p=0.116) staining. C. Quantification of the percent colocalization of α2 with VGAT comparing *wildtype* (11.20 ± 1.264), with *Gabra2*-1 *heterozygous* (7.72 ± 0.805) and *homozygous* (7.22 ± 0.867; p=0.018) staining. Scale bar = 10 µm. All plots shown and all values listed are mean ± standard error.

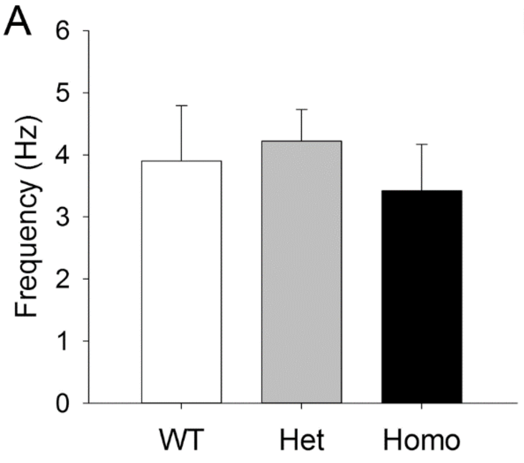

B

	Control		Het			Homo		
	Mean	SE	Mean	SE	p	Mean	SE	p
sIPSC amplitude (pA)	48.6	1.12	39.2	0.85	<0.001	40.1	0.65	<0.001
sIPSC frequency (Hz)	3.9	0.89	4.22	0.51	0.82	3.42	0.75	0.68
	n=10 cells From 5 mice		n=13 cells From 6 mice			N=8 cells From 5 mice		
sIPSC Decay (s)	4.38	0.12	3.88	0.09	0.001	3.39	0.13	<0.000 1
	n=200 events From 5 mice		n=200 events From 6 mice			n=200 events From 5 mice		

Supplementary Figure 7. Electrophysiological assessment reveals no difference in frequency in *Gabra2*-1 slices. A. Quantification of sIPSC frequency in *wildtype*, *heterozygous*, and *homozygous Gabra2*-1 mice. B. Summary table of sIPSC parameters. All plots shown and all values listed are mean ± standard error.

Supplementary Table 4. Modified SHIRPA screen results summary

Modality/Assessment		Wildtype	Het	Homo
**Viability**		Normal	Proportion die (PND15-21)	Proportion die (PND15-21)
**Visual:**				
Corneal Reflex		Normal	Normal	Normal
Visual Placing		Normal	Normal	Normal
**Olfactory:**				
Buried Food Retrieval		Normal	Normal	Normal
**Auditory:**				
Pinna Reflex		Normal	Normal	Normal
Startle Response (120 dB pulse)		0.943±0.306	0.889±0.529	0.886±0.192
**Rotarod** (rpm)	18	54.0±6.0	58.8±1.9	
	24	53.8±6.3	56.5±2.6	
	28	56.8±2.4	55.6±3.2	

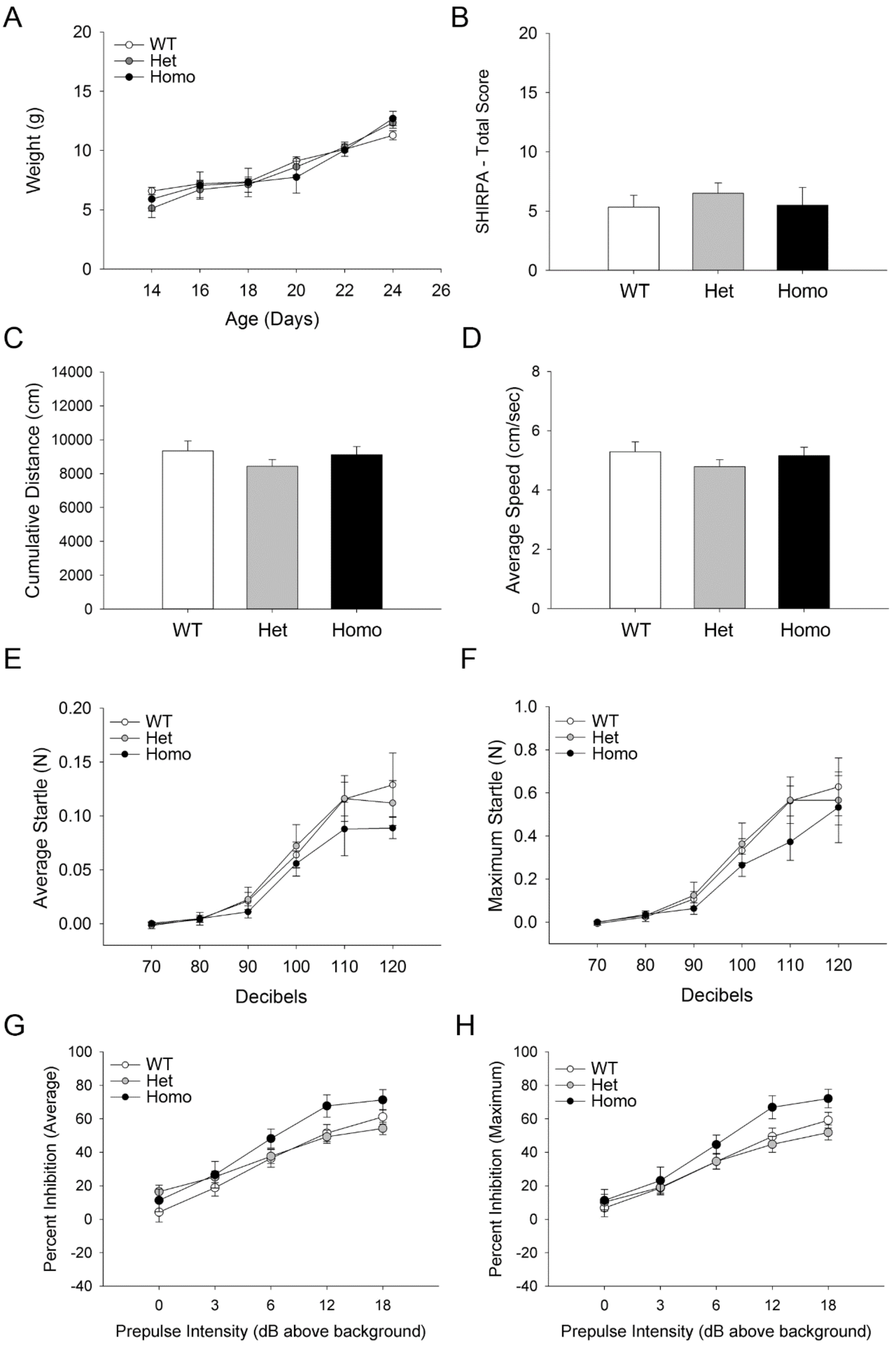

Supplementary Figure 8. An overall health screen in *Gabra2*-1 mice revealed no difference from *wildtype* control. A. Pre-weaning weight gain. B. SHIRPA total score. C,D. Cumulative distance and average speed in the open field. E,F. Average and maximum startle response. G,H. Average and maximum pre-pulse inhibition. All plots shown and all values listed are mean ± standard error.

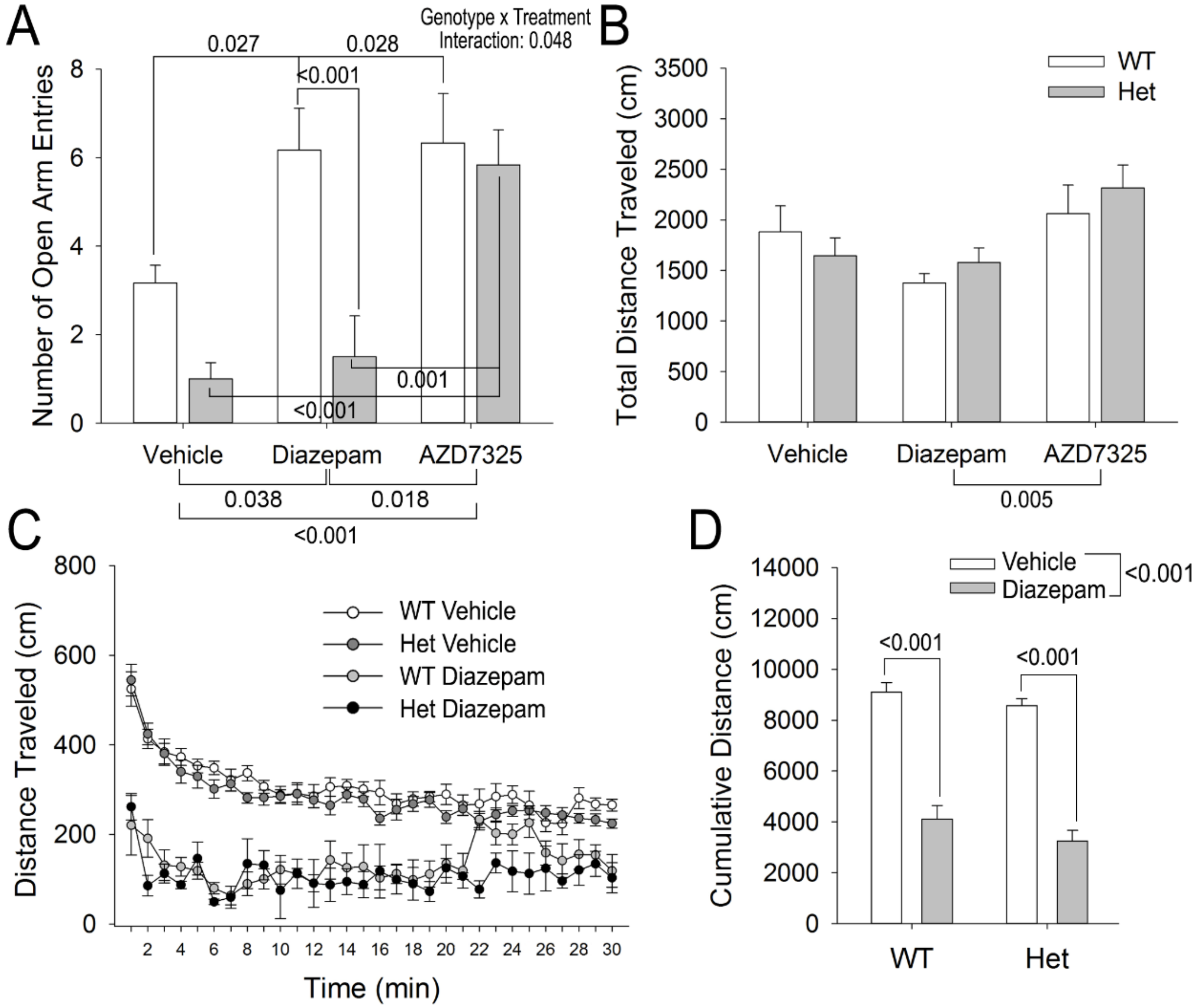

Supplementary Figure 9. Diazepam induced sedation does not differ between *Gabra2*-1 and *wildtype* controls. A. Analysis of arm entries in the elevated plus maze comparing *wildtype* (LS Mean 5.22), *Gabra2*-1 *heterozygous* (LS Mean 2.78; SE of LS Mean – 0.466) mice treated with vehicle (LS Mean 2.083), diazepam (LS Mean 3.833), or AZD7325 (LS Mean 6.08; SE of LS Mean – 0.571). B. Analysis of total distance traveled in the elevated plus maze comparing *wildtype* (LS Mean 1773.55), *Gabra2*-1 *heterozygous* (LS Mean 1847.32; SE of LS Mean – 119.271) mice treated with vehicle (LS Mean 1746.07), diazepam (LS Mean 1477.44), or AZD7325 (LS Mean 2189.79; SE of LS Mean – 146.077). C. Distance travelled in the open field following diazepam injection (or vehicle control) comparing *wildtype* and *Gabra2*-1 *heterozygous* mice. B. Cumulative distance traveled comparing diazepam (LS Mean 3680.54) to vehicle control (LS Mean 8838.16) in *wildtype* (LS Mean 6607.37) and *Gabra2*-1 *heterozygous* (LS Mean 5911.34) mice (genotype p=0.137; treatment p<0.001). All plots shown and all values listed are mean ± standard error.

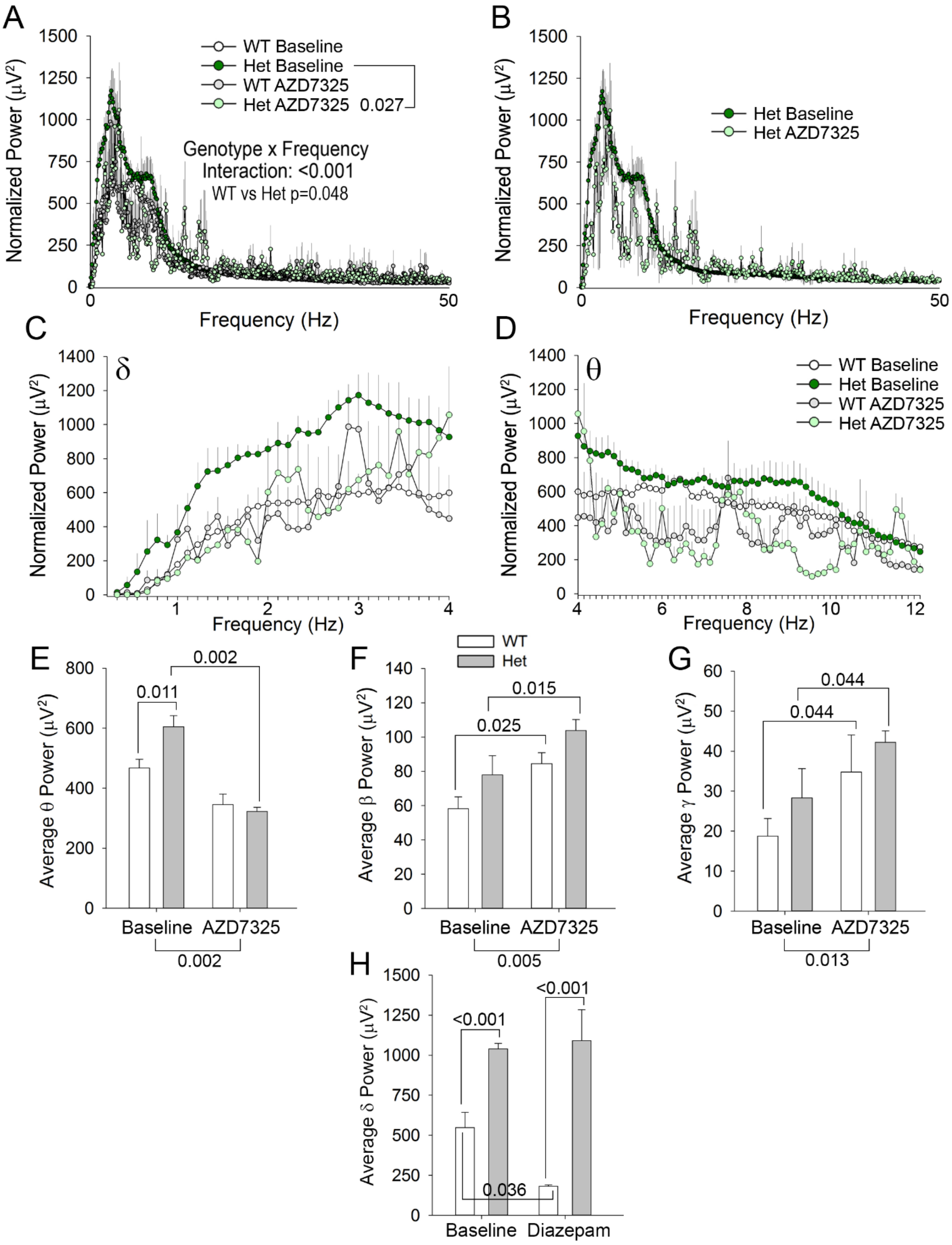

Supplementary Figure 10. EEG recordings of *Gabra2*-1 *heterozygous* mice compared to *wildtype* controls. A. Cumulative FFT comparing *wildtype* and *Gabra2*-1 *heterozygotes* under baseline and AZD7325 treated conditions. B. Cumulative FFT comparing baseline and AZD7325 treatment in *Gabra2*-1 *heterozygous* mice. Zoom in on the cumulative FFT for δ frequencies (C; 0.25-4 Hz) and θ frequencies (D; 4-12 Hz). Average power in θ (E; 4-12 Hz; LS Mean WT 406.57 ± 17.044; LS Mean Het 463.84 ± 14.761; LS Mean

baseline 536.11; LS Mean AZD7325 334.30; genotype p=0.052; treatment p=0.002), β (F; 12-30 Hz; LS Mean WT 71.37 ± 8.201; LS Mean Het 90.89 ± 7.102; LS Mean baseline 68.06; LS Mean AZD7325 94.20; genotype p=0.132; treatment p=0.005), γ (G; >30 Hz; LS Mean WT 26.77 ± 6.043; LS Mean Het 35.28 ± 5.233; LS Mean baseline 23.55; LS Mean AZD7325 38.50; genotype p=0.336; treatment p=0.013) frequencies comparing *wildtype* and *Gabra2*-1 *heterozygous* mice under baseline conditions and following treatment with AZD7325. H. Average power in δ frequencies (0.25-4 Hz; LS Mean WT 364.55; LS Mean Het 1065.47; LS Mean baseline 793.47; LS Mean diazepam 636.55; genotype p<0.001; treatment p=0.146) comparing *wildtype* and *Gabra2*-1 *heterozygous* mice under baseline conditions and following treatment with diazepam. All plots shown and all values listed are mean ± standard error.

Supplementary Table 5. Statistical Analyses.

Figure Pannel	Groups	Mean	SE	F	Main Effects	Multiple Comparisons
1J	α2<br>α2-1	1.0000<br>0.6894	0<br>0.08220		0.048	
2E loop	WT<br>Het<br>Homo	1.505<br>0.671<br>-0.225	0.0606<br>0.0112<br>0.0165	551.243	<0.001	WT vs Het <0.001<br>WT vs Homo <0.001<br>Het vs Homo <0.001
2E c-term	WT<br>Het<br>Homo	0.592<br>0.711<br>1.012	0.0383<br>0.0262<br>0.0330	54.999	<0.001	WT vs Het 0.002<br>WT vs Homo <0.001<br>Het vs Homo <0.001
2E Geph	WT<br>Het<br>Homo	1.410<br>1.357<br>1.433	0.0933<br>0.0612<br>0.1260	0.164	0.853	
2E CB	WT<br>Het<br>Homo	0.560<br>0.276<br>0.292	0.0681<br>0.0728<br>0.0488	6.180	0.011	WT vs Het 0.018<br>WT vs Homo 0.010<br>Het vs Homo 0.859
3B	WT<br>Het<br>Homo	0.365<br>0.279<br>0.271	0.0040<br>0.0028<br>0.0030			WT vs Het <0.001<br>WT vs Homo <0.001
3C	WT<br>Het<br>Homo	192.8<br>202.7<br>347.2	7.851<br>17.490<br>5.276	25.3	0.001	WT vs Homo <0.001<br>Het vs Homo <0.001
3D	WT<br>Het<br>Homo	145.00<br>184.10<br>165.00	17.50<br>6.31<br>28.33	1.32	0.3102	
3H	WT<br>Het<br>Homo	0.344<br>0.232<br>0.188	0.0242<br>0.0350<br>0.0199	9.791	<0.001	WT vs Het 0.011<br>WT vs Homo <0.001<br>Het vs Homo 0.301
3I	WT<br>Het<br>Homo	3056.056<br>1913.262<br>1087.936	338.296<br>275.019<br>165.428	10.789	<0.001	WT vs Het 0.025<br>WT vs Homo <0.001<br>Het vs Homo 0.100
3J	WT<br>Het<br>Homo	3447.821<br>3165.590<br>3346.526	250.004<br>390.816<br>289.297	0.210	0.812	
5D	WT<br>Het<br>Homo	26.050<br>15.340<br>10.40	2.123<br>2.057<br>1.241	18.662	<0.001	WT vs Het <0.001<br>WT vs Homo <0.001<br>Het vs Homo 0.070
5E	WT<br>Het<br>Homo	27.640<br>22.90<br>24.73	3.302<br>2.993<br>2.739	0.627	0.542	
5G	WT<br>Het	7.108<br>2.385	0.260<br>0.188	223.345	<0.001	WT vs Het <0.001<br>WT vs Homo <0.001

	Homo	1.112	0.182			Het vs Homo <0.001
6D	WTxWT	3.200	3.200	18.362	<0.001	
	WTxHet	69.048	8.292			
	HetxHet	77.379	4.494			
	HetxHomo	90.910	9.090			
	HomoXHomo	100.000	0.000			
6E	WTxWT	97.778	2.222	7.024	<0.001	
	WTxHet	60.630	4.979			
	HetxHet	63.569	4.416			
	HetxHomo	46.409	14.130			
	HomoXHomo	36.498	2.212			
6G	WT	0.740	0.401	Genotype – 20.346	<0.001	WT vs Het 0.002
	Het	4.190		Time – 15.892	<0.001	WT vs Homo <0.001
	Homo	3.400		Interaction – 7.019	<0.001	Het vs Homo 0.197
6I	WT	58.160	3.308	6.178	0.010	WT vs Het 0.007
	Het	33.392	5.524			WT vs Homo 0.010
	Homo	31.085	4.527			Het vs Homo 0.732
6J	WT	9.443	0.973	0.484	0.631	
	Het	9.948	2.523			
	Homo	12.387	2.836			
6K	WT	143.075	13.048	8.980	0.002	WT vs Het 0.010
	Het	390.707	88.210			WT vs Homo 0.001
	Homo	501.918	57.633			Het vs Homo 0.214
7A	WT	104.523	11.114	3.962	0.027	WT vs Het 0.030
	Het	71.770	6.890			WT vs Homo 0.034
	Homo	73.500	10.337			Het vs Homo 0.895
7B	WT	25.224	4.866	6.686	0.005	WT vs Het 0.007
Open	Het	10.948	1.899			WT vs Homo 0.008
	Homo	8.225	2.954			Het vs Homo 0.602
7B	WT	74.776	4.866	6.686	0.005	WT vs Het 0.007
Closed	Het	89.052	1.899			WT vs Homo 0.008
	Homo	91.775	2.954			Het vs Homo 0.602
7C	WT	31.581	1.508	Genotype – 28.996	<0.001	WT vs Het <0.001
	Het	20.096				
	Vehicle	15.292	1.847	Treatment – 45.335	<0.001	Veh vs Diaz 0.008
	Diazepam	22.670				Veh vs AZD <0.001
	AZD7325	39.552				Diaz vs AZD <0.001
				Interaction – 17.243	<0.001	
7F	WT	82.023	8.227	Genotype – 7.893	0.038	
	Het	112.598	7.125	Frequency – 77.475	<0.001	
				Interaction – 3.529	<0.001	
7G	WT	450.284	47.627	Genotype – 6.784	0.048	WT vs Het 0.048
	Het	614.384	41.246			
				Treatment – 2.863	0.151	Base vs AZD 0.151
	Baseline	598.931	50.416			
	AZD7325	465.738		Interaction – 5.674	0.063	Within WT 0.667
						Within Het 0.027
						Within baseline 0.006
						Within AZD7325 0.821
