# Supplementary material for: Developmental seizures and mortality result from reducing GABAA receptor α2-subunit interaction with collybistin
Source: Nat Commun. 2018 Aug 7;9:3130. doi: 10.1038/s41467-018-05481-1 (PMC6081406; doi:10.1038/s41467-018-05481-1)
Supplement: Supplementary file 3 — Description of Additional Supplementary Files [file 41467_2018_5481_MOESM3_ESM.pdf]

Description of Additional Supplementary Files

File Name: Supplementary Movie 1

Description: Observations of spontaneous seizure in Gabra2-1 pups. Wildtype littermate pups are shown first, followed by example recordings of Gabra2-1 pups found seizing.
